# Plasticity of circulating tumor cells in small cell lung cancer

**DOI:** 10.1038/s41598-023-38881-5

**Published:** 2023-07-21

**Authors:** Jiyoun Seo, Mihir Kumar, Jeremy Mason, Fiona Blackhall, Nicholas Matsumoto, Caroline Dive, James Hicks, Peter Kuhn, Stephanie N. Shishido

**Affiliations:** 1 Convergent Science Institute in Cancer, Michelson Center for Convergent Bioscience, University of Southern California, Los Angeles, CA 90089 USA; 2 Institute of Urology, Catherine & Joseph Aresty Department of Urology, Keck School of Medicine, University of Southern California, Los Angeles, CA 90033 USA; 3 Keck School of Medicine, Norris Comprehensive Cancer Center, University of Southern California, Los Angeles, CA 90033 USA; 4 Department of Medical Oncology, The Christie NHS Foundation Trust, Manchester, UK; 5 Cancer Research UK Lung Cancer Centre of Excellence, University of Manchester and University College London, Manchester, UK; 6 CRUK Manchester Institute Cancer Biomarker Centre, University of Manchester, Manchester, UK; 7 Department of Biological Sciences, Dornsife College of Letters, Arts, and Sciences, University of Southern California, Los Angeles, CA 90089 USA; 8 Department of Aerospace and Mechanical Engineering, Viterbi School of Engineering, University of Southern California, Los Angeles, CA 90089 USA; 9 Department of Biomedical Engineering, Viterbi School of Engineering, University of Southern California, Los Angeles, CA 90089 USA

**Keywords:** Mechanisms of disease, Small-cell lung cancer

## Abstract

Small cell lung cancer (SCLC) is an aggressive neuroendocrine tumor with low five-year survival rates. Recently described molecular phenotypes of SCLC exhibit differential vulnerabilities heralding potential for stratified treatment. Whilst tumor biopsy in SCLC is challenging, circulating tumor cells in the liquid biopsy are prevalent and can be repeatedly sampled accommodating the dynamic plasticity of SCLC phenotypes. The aim of this study was to characterize the heterogeneity of rare circulating cells with confirmed tumor origin and to explore a liquid biopsy approach for future clinical trials of targeted therapies. This study applied the 3rd generation of a previously validated direct imaging platform to 14 chemo-naive SCLC patients and 10 non-cancerous normal donor (ND) samples. Phenotypic heterogeneity of circulating rare cells in SCLC was observed and a patient-level classification model was established to stratify SCLC patients from non-cancerous donors. Eight rare cell groups, with combinations of epithelial, endothelial, and mesenchymal biomarker expression patterns, were phenotypically characterized. The single-cell genomic analysis confirmed the cancer cell plasticity in every rare cell group harboring clonal genomic alterations. This study shows rare cell heterogeneity and confirms cellular plasticity in SCLC providing a valuable resource for better opportunities to discover novel therapeutic targets in SCLC.

## Introduction

Small cell lung cancer (SCLC) is an aggressive and rapidly metastasizing neuroendocrine malignancy with an average 5-year survival rate of 7%^1^. SCLC comprises about 10–15% of all lung cancers with the remainder classified as non-small cell lung cancer (NSCLC)^2^. Despite affecting a smaller fraction of patients, the survival rates of patients with SCLC are significantly lower than those with NSCLC. For SCLC patients with limited disease, the 5-year survival rate is 20%, while the 5-year survival rate for those with extensive SCLC is < 1%. Given the large decrease in survival rate for extensive disease compared to limited disease, it is critical to identify diagnostic and prognostic biomarkers to detect and monitor the disease at its earliest stage to assist in treatment decision-making when it could be most effective.

Despite the aggressive nature of SCLC, the standard of care for first- and second-line therapies has not appreciably changed for nearly four decades^3^. While SCLC initially responds to chemotherapy and radiation therapy, there is a rapid emergence of relapse and drug resistance leading to short survival times in the majority of patients^4–6^. Novel treatment approaches have shown promise, especially with immune checkpoint inhibitors combined with platinum and etoposide^4^. However, robust and sustained responses to those regimens have not been demonstrated. This supports the need for new approaches to better monitor treatment efficacy and to better characterize the disease for improved management of metastatic SCLC.

Recent studies exhibit the characterization of molecular phenotypes of SCLC, highlighting tumor heterogeneity and cellular plasticity for potential treatment stratification and the development of targeted therapies^3,7,8^. Phenotype-determined cellular plasticity is an important phenomenon in cancer progression, emerging as a contributor to therapy evasion^9–11^. Cellular plasticity allows a cancer cell to change phenotype without additional genetic mutations, which may be independent of therapeutic pressure. Studies in various cancer types have shown that a neoplastic cell can hijack developmental processes as a way to adapt to environmental stressors^8,11^.

Epithelial-to-mesenchymal transition (EMT) is one of the well-known examples of showing plasticity which consists of both morphological and molecular changes. Vascular mimicry (VM) is another example where cancer cells trans-differentiate and acquire endothelial cell behavior. We have previously shown that VM cells represented within circulating tumor cells (CTCs) in SCLC enable the de novo generation of vascular networks which could contribute to dissemination and metastasis^12^. Furthermore, cellular plasticity allows the conversion of cells between four defined subtypes of SCLC cells with distinct therapeutic vulnerabilities previously classified based on the differential expression of four biomarkers: ASCL1, NEUROD1, POU2F3, and YAP1^7,11,13^. The ability to profile a tumor at this deep molecular level will deliver personalized and more stratified treatment options for this aggressive neuroendocrine cancer.

One major barrier to understanding SCLC has been the limited access to tissue samples for a comprehensive analysis of the disease. This is due to SCLC patients rarely undergoing surgical resection and even then only limited numbers of cells are available^14^. A liquid biopsy approach can provide a minimally invasive route to repeatedly detect clinically relevant analytes^14–16^. CTCs detected in the liquid biopsy of SCLC patients have been confirmed as a prognostic biomarker with the potential to improve therapeutic strategies^3,12,17^. The liquid biopsy can characterize the disease at the single-cell level and resolve the limitations of tissue biopsy, allowing for routine, non-invasive sampling.

In this study, we apply a third-generation advanced direct imaging platform (high-definition single-cell assay; HDSCA3.0) to analyze the liquid biopsy^17^ and identify the phenotypic and genotypic heterogeneity of circulating rare cells in SCLC patients. We investigated various cell groups that are differentially observed in SCLC patients allowing for stratification from non-cancerous donors (NDs). Single-cell genomic analysis revealed the clonal populations and confirmed the SCLC cellular plasticity of CTCs detected by the liquid biopsy. The data presented provides the molecular characterization of a wide spectrum of CTC subtypes detected in SCLC patient samples.

## Results

This study consists of 24 peripheral blood samples collected from 14 chemo-naive SCLC patients and 10 NDs. One test of a sample consists of two slides being analyzed, therefore a total of 28 slides (2,304,659 cells/slide on average) from SCLC patients and 20 slides (2,116,477 cells/slide on average) from NDs were used.

### Patient-level classification modeling

**Figure 1:**
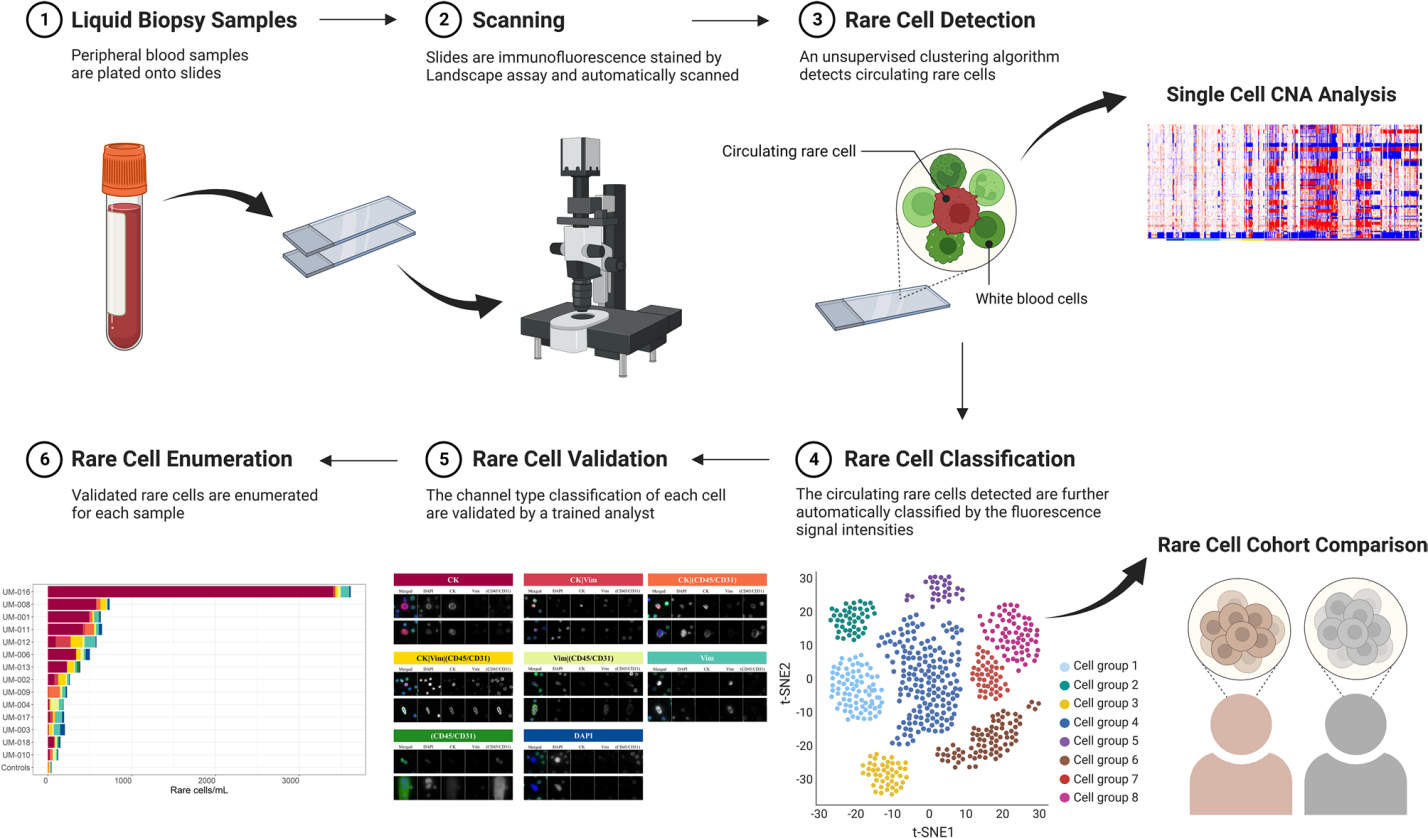
HDSCA3.0 Workflow. (1) Peripheral blood samples are plated onto slides. (2) Slides are immunofluorescence stained by Landscape assay and automatically scanned. (3) Detection of rare cells from the imaging data set is conducted using an unsupervised clustering algorithm that clusters rare cells using extracted quantitative morphologic features. Further downstream analysis of single-cell copy number alteration (CNA) on those detected rare cells is performed. (4) The circulating rare cells detected are further automatically classified into channel-based cell classifications defined by the fluorescence signal intensities. (5) The channel-based cell classification of each cell is validated by a trained analyst. (6) Enumeration of each rare cell type per sample is counted.

We first investigated whether the liquid biopsy could differentiate between SCLC patients and NDs by exploring the differential cellular populations. A patient-level classification model was constructed using the rare cell populations detected by HDSCA3.0. Figure 1 shows a schematic of the overall data science pipeline. It is important to note that NDs often exhibit small numbers of rare cells across the range of channel types^18^, making a rigorous statistical treatment necessary to distinguish SCLC from ND.

**Figure 2:**
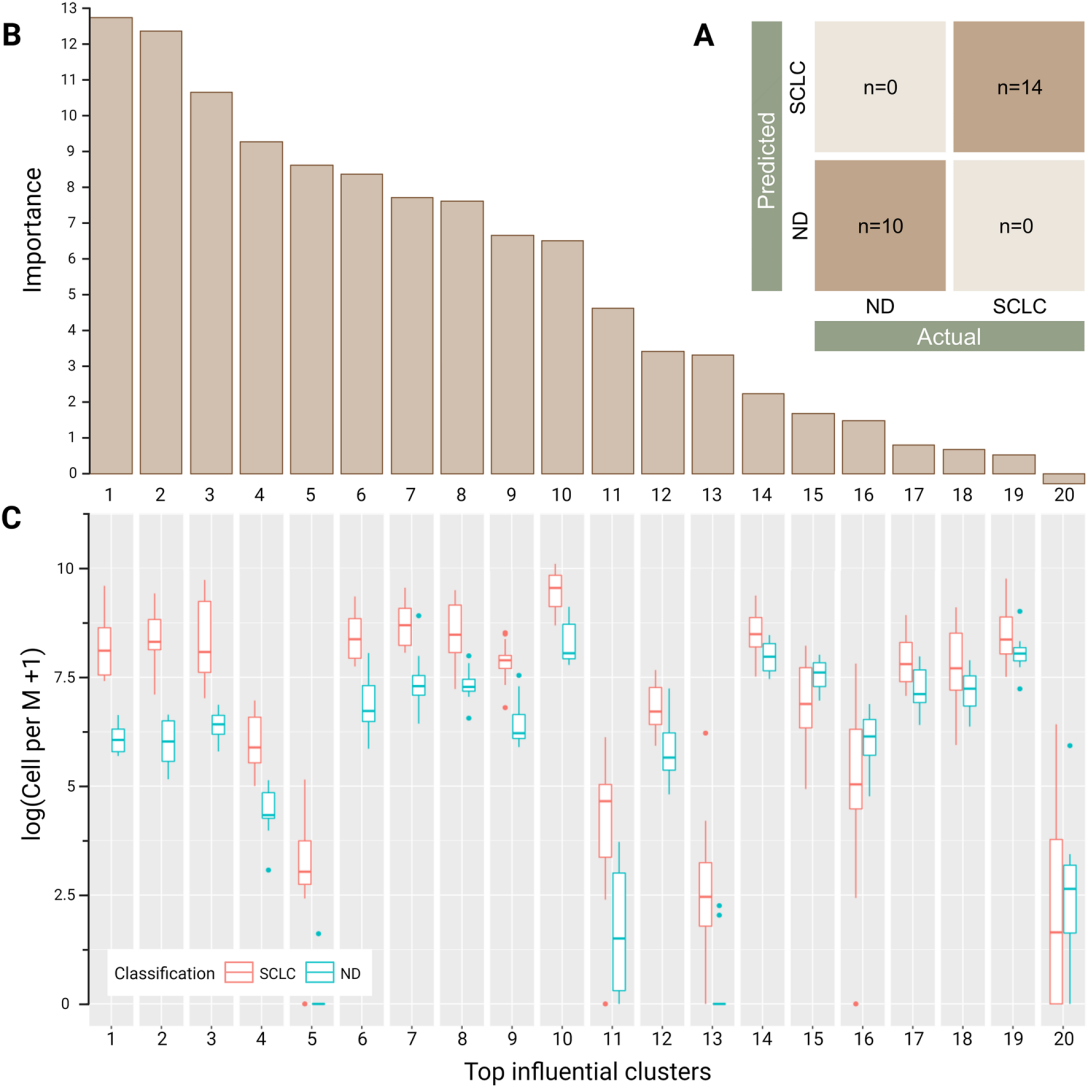
Patient-level classification model results using HDSCA3.0 liquid biopsy data. (**A**) Confusion matrix of predicting NDs and SCLC patients. (**B**) Feature importance ranking of the top influential clusters. Depicts the relative contribution of each event cluster to making correction predictions. (**C**) Box plots showing the distribution of the top 20 influential clusters separated by each classification. The plot is shown on a logarithmic scale depicting cells per million (Cell per M).

The patient-level classifier for the given prediction problem of differentiation between SCLC patients and NDs showed perfect concordance (100%) with correct predictions across all patients (Fig. 2A). This verifies that the rare cells in SCLC detected by HDSCA3.0 have a significant influence in differentiating SCLC patients from NDs. The top-most influential cellular clusters that had the highest impact on stratification (Fig. 2B) were further investigated to examine what phenotypic cellular populations comprise them. Figure 2C shows the distributions of rare cells for each cluster. The top 14 groups all showed higher counts within the SCLC group as compared to the NDs. Interestingly, the most important clusters consisted of various cellular phenotypes including the pan-cytokeratin (CK)-positive CTC candidates previously described in SCLC patients^17^ and also cell phenotypes that have not previously been described in SCLC (Fig. S1). This patient-level classification modeling of the liquid biopsy indicates that (1) a peripheral blood sample can stratify SCLC from ND, and (2) a heterogeneous population of rare cells exists in SCLC which are influential in differentiation between SCLC patients and NDs.

### Characterization of CTCs

**Figure 3:**
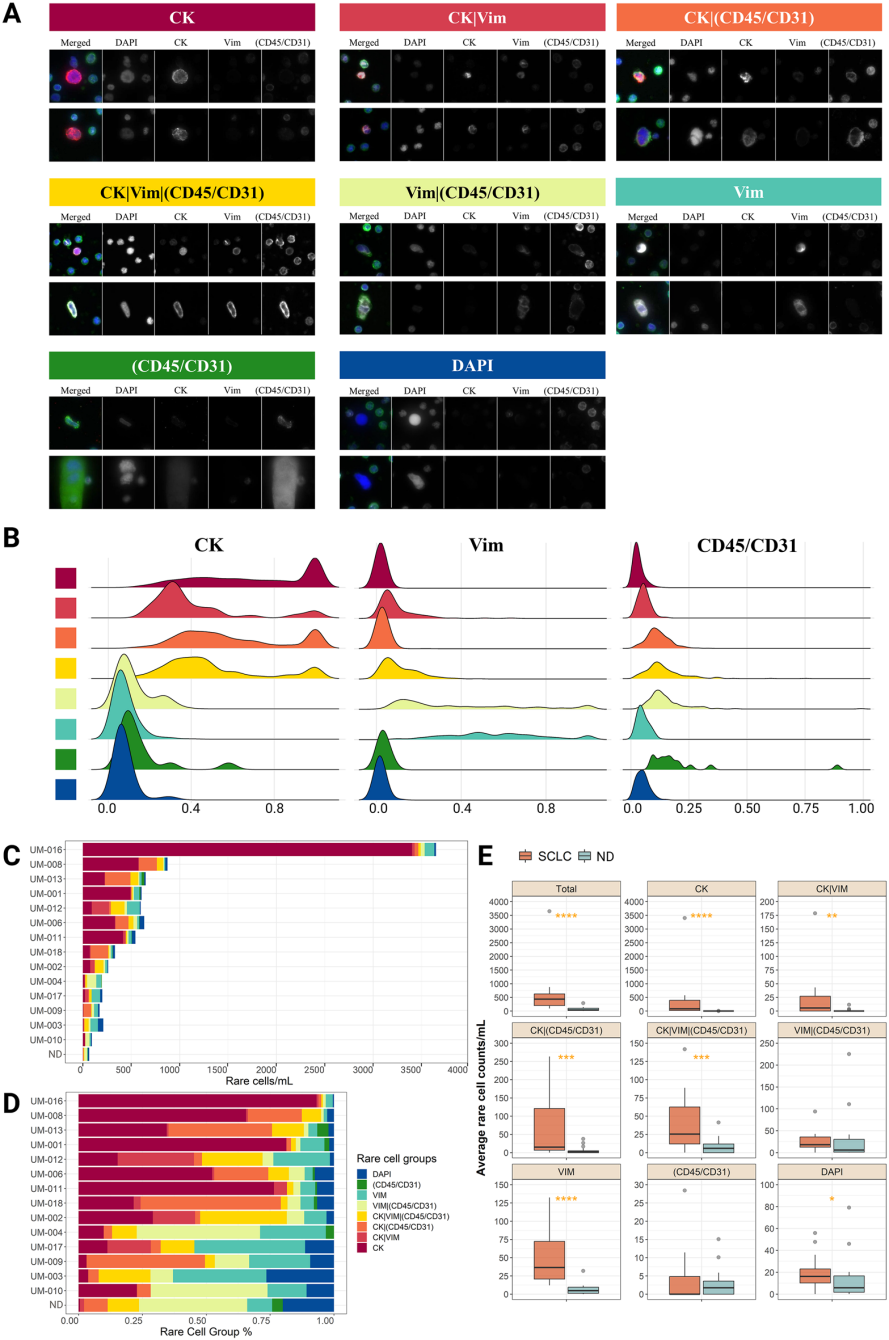
Circulating rare cells identified in the peripheral blood from SCLC patient samples using HDSCA3.0 with the Landscape assay. (**A**) Representative cell images of each cell group. Each row shows a composite image plus each of the four channels separately; DAPI in blue, CK in red, Vim in white, (CD45/CD31) in green. (**B**) Signal distribution of immunofluorescent markers for channel-based cells. Each cell group is correspondingly annotated to (**A**) with eight different colors. (**C**) The number of total rare cells per ml is calculated for each SCLC patient and ND. Each rare cell group is annotated with eight different colors which are shown in the bottom middle of the figure. The number of rare cells of 50 NDs was averaged. (**D**) Proportion plot of the rare cell groups of each SCLC patient and averaged NDs. (**E**) Box plots comparing cell counts per ml of each cell group between NDs and SCLC patient samples. *: *p-value* ≤ 0.05, **: *p-value* ≤ 0.01,***: *p-value* ≤ 0.001,****: *p-value* ≤ 0.0001.

We further examined the phenotypic cellular populations in each patient using manual channel-type classifications. Figures 3A and S2 show representative single-cell images of each channel-based classification detected in the liquid biopsy of SCLC patients. The signal distribution of CK, Vim, and CD45/CD31 immunofluorescent markers for channel-based cells is shown in Fig. 3B. The enumeration and proportion of the eight different channel-based cell groups in each SCLC patient and the NDs are shown in Fig. 3C and D. The CK CTCs were detected significantly more in SCLC patients (mean: 411.19 cells/ml, range: 5–3402.05 cells/mL) compared to the NDs (mean: 0.35 cells/ml, range: 0–3.77 cells/ml, *p-value* < 0.0001). The CK CTCs accounted for over 50% of total circulating rare cells from 43% of SCLC patients (n = 6). Interestingly, not only CK only CTCs but also CK|Vim CTCs were detected significantly more in SCLC (mean: 23.82, range: 0–178.69 cells/ml) compared to the NDs (mean: 1.03, range: 0–11.47 cells/ml, *p-value* = 0.046) as well as most of the other circulating rare cell groups (Fig. 3E). In general, the enumeration of total rare cells was significantly greater in the SCLC patient cohort (mean: 602.39 cells/ml) compared to the ND cohort (mean: 65.67 cells/ml, *p-value* < 0.0001).

**Figure 4:**
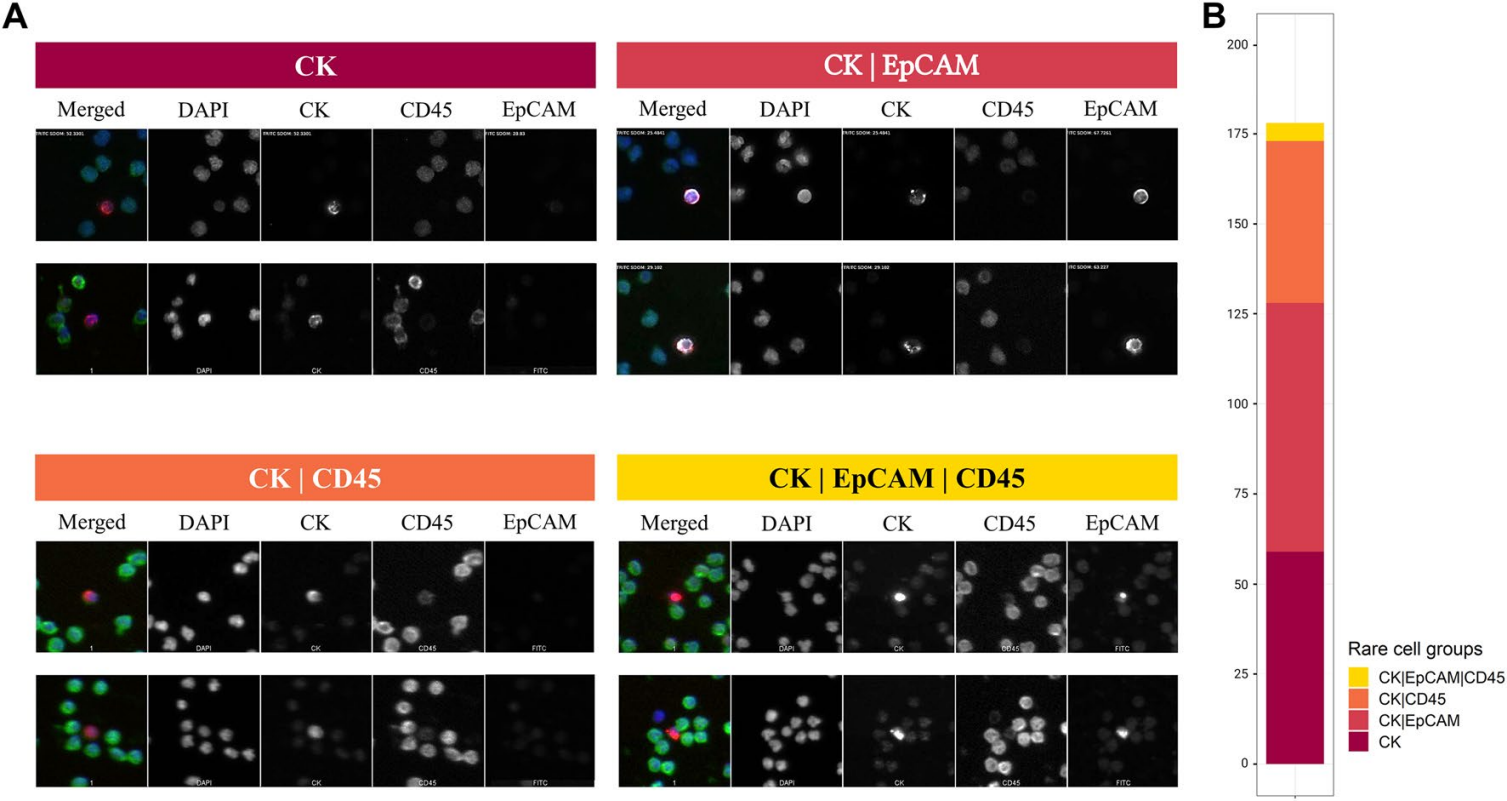
Detected cells from UM-001 using the EpCAM assay. (**A**) Representative cell images of each cell group. Each row shows a composite image plus each of the four channels separately: DAPI in blue, CK in red, CD45 in green, and EpCAM in white. (**B**) The number of total rare cells per mL is provided. Each rare cell group is annotated with four different colors which are shown in the bottom right of the figure.

To further investigate the modulation of EpCAM expression in the CTC population in SCLC, the HDSCA workflow was conducted using the EpCAM-targeted assay on one SCLC patient (UM-001). Figure 4A shows the images of representative cells of each channel-based classification in which the majority of cells were identified as CK CTCs and CK|EpCAM CTCs (71.9%, 128 out of 178 cells). Interestingly, we detected the modulation of EpCAM expression; from EpCAM-positive to EpCAM-negative cells within the CTCs. A total of 69 CK|EpCAM CTCs and 59 CK CTCs were detected. We further detected the presence of CK|CD45, and CK|EpCAM|CD45 cells (Fig. 4B). Together, this case study demonstrates the presence of a heterogeneous phenotypic population of CTCs in SCLC.

### Genome-wide single-cell copy number analysis

Single-cell genomic analysis was conducted to determine whether the detected CTC candidates and other rare cell types exhibit clonal genomic alterations characteristic of SCLC. Single-cell copy number alteration (CNA) profiling was performed on 309 cells in total from 14 SCLC patients: 139 CK CTCs, 11 CK|Vim CTCs, 26 CK|Vim|(CD45/CD31) cells, 27 CK|(CD45/CD31) cells, 40 Vim cells, 24 Vim|(CD45/CD31) cells, 14 CK|EpCAM CTCs, and 21 DAPI cells. White blood cells were also isolated from each patient as internal controls. Figure S3 shows a heatmap of single-cell CNA profiles of isolated rare cells from each of the 14 SCLC patients, clustered by each channel-based classification. Interestingly, genomic alterations were observed in seven types of CTC candidates with various phenotypic combinations of epithelial, endothelial, and mesenchymal biomarker expression, not only in the CK CTCs.

**Figure 5:**
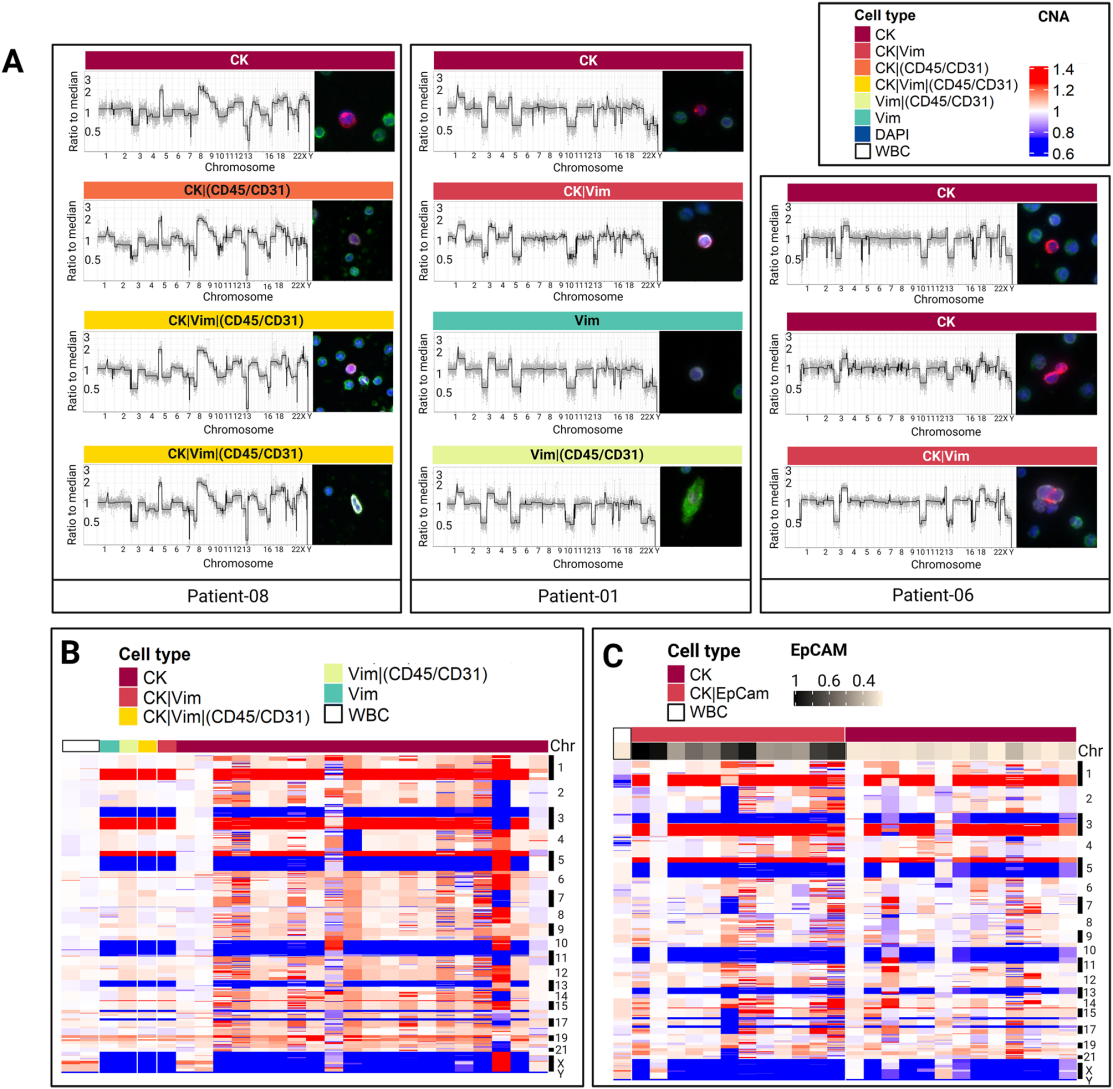
Single-cell CNA profiling of rare cells detected in SCLC peripheral blood samples. (**A**) CNA profile of representative single rare cells from each SCLC patient and their Landscape-stained cell images; CK in red, Vim in white, CD45/CD31 in green, and DAPI in blue. The rare cell type of each cell is annotated with color labels at the top right. (**B**) CNA profiles of sequenced cells from UM-001 stained by Landscape assay. (**C**) CNA profiles of sequenced cells from UM-001 stained by EpCAM-targeted assay. EpCAM expression for each cell is shown at the bottom of the heatmap. The rare cell group of each cell in B and C are annotated with different color labels at the top left of each heatmap. CNA gains are shown in red, neutrals in white, and losses in blue.

Furthermore, the presence of a genetically clonal CTC population that is highly phenotypically variable confirmed cellular plasticity (Fig. 5A). CK CTCs, CK|(CD45/CD31), and CK|Vim|(CD45/CD31) cells in Patient 8 harbored clonal gene losses in tumor suppressor genes such as RB1, TP53, and PTEN that are known as the most frequently altered genes in SCLC^19,20^. Loss of one copy of chromosome 3p is one of the most frequent and early events in human cancer^21^. Gains of 8q containing the MYC gene that has been identified as an oncogenic driver in SCLC^21^ and including the RICTOR gene, a subunit of the mTORC2 complex, as well as in the IL7R gene were observed. CK CTCs, CK|Vim cells, and CK|Vim|(CD45/CD31) cells in Patient 6 also harbored clonal alterations in SCLC-associated genes (Figs. 5A and S3), including the tumor suppressor genes, PTEN, RB1, and TP53. The heatmaps for all of the patients are shown in Fig. S3.

In addition to the Landscape assay, Patient 1 was further analyzed for EpCAM. Figure 5B and C show Patient 1 with analysis of cells isolated from both the Landscape and EpCAM-targeted assays. The clonal population had losses in the 3p, 10q, 13q, and 17p regions corresponding to RASSF1, PTEN, RB1, and TP53, respectively. The gains associated with the clonal population of cells were identified in 1q, 3q, and 5p regions corresponding to BCL9, MUC1 (1q), PIK3CA (3q26), p63 (3q28), and TERT (5p15). These CNAs have been confirmed from recent studies to be recurrently lost in SCLC^22–25^. The clonal alterations were observed in 16 out of 20 (80%) CK CTCs, one CK|Vim cell, one CK|Vim|(CD45/CD31) cell, one Vim cell, and one Vim|(CD45/CD31) cell from the Landscape assay. The images of those cells from the Landscape staining are shown in Fig. 5A. Across the range of CK and EpCAM expression, 11 out of 12 (91%) CK|EpCAM CTCs and 10 out of 13 (77%) CK CTCs exhibit low or no expression of EpCAM and also shared clonal alterations.

Together, CNA analysis (1) verifies that the CK and CK|EpCAM CTCs have clonal alterations, (2) displays CTC heterogeneity, and (3) confirms CTC plasticity.

### Single-cell alteration classification model

**Figure 6:**
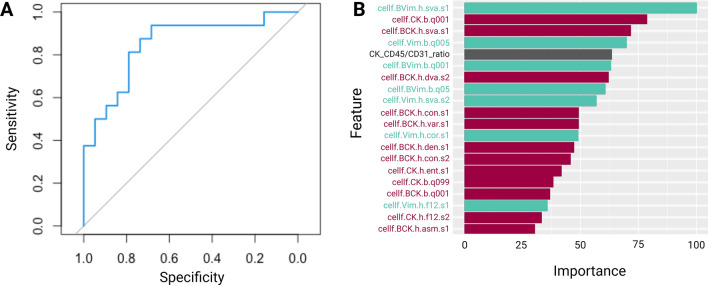
Single-cell alteration classification model performance. (**A**) ROC Curve of the random forest classification model. (**B**) Feature importance of the top 20 features used in the model. The whole list of features is shown in Fig. S4.

An association between phenotypic characteristics and genomic alteration was investigated to assess the significance of phenotypic variability in SCLC. The random forest classification model using the phenotypic features of the rare cells was conducted to classify each rare cell as clonally altered or not. The quantitatively extracted cellular phenotypic features include the intensity of the immunofluorescence markers and morphometric characteristics. The classifier showed a high performance of 0.86 Area Under the receiver operating characteristic Curve (AUC) score (Fig. 6A). The importance of the features was calculated to investigate the significance of different phenotypic features in predicting genomic alterations (Fig. 6B).

12 of the top 20 features for the model were CK-related features that help the model identify the genomically clonal, altered, SCLC cells (Fig. 6B). Such features included the ratio of CK to CD45/CD31 positivity and the shape or size of the CK positivity within the cell. In addition to the CK-related influential features, Vim-related phenotypic features were also counted to strongly affect the classification of clonal SCLC cells (7 out of the top 20 features). Overall, our classification model of single-cell alteration showed a robust performance utilizing the cellular phenotypic features and the characteristics of intensity or morphologies of CK and Vim expression were the strongest predictors.

## Discussion

In this study, we describe several important findings in SCLC: (1) SCLC patients and NDs can be stratified using a liquid biopsy, (2) detection of a heterogeneous population of CTCs, and (3) characterization of SCLC cellular plasticity.

A patient-level classification model was able to stratify the SCLC patients from NDs with perfect concordance using the rare cells detected by HDSCA3.0, confirming the abundance of circulating rare cells as a clinically useful analyte for SCLC patients. Furthermore, the rare cells comprising the most important clusters included not only the CK-positive cells previously identified as CTCs^3,26^ but also a phenotypically various cellular population not previously described for stratifying SCLC patients from NDs. This emphasizes the power of a rare cell framework in detecting ultra-rare CTCs and their potential utility as a complementary tool to current methods of imaging and pathology tests for the diagnosis of SCLC.

We investigated the phenotypic heterogeneity of CTCs in SCLC through the utilization of multiple assays to characterize a wide spectrum of rare cells. Through the utilization of multiple epithelial biomarkers in the EpCAM-targeted assay, we observed the wide range of CK and EpCAM expression in the CTC population in which 46% of the CTC population did not express EpCAM. We have previously shown that the HDSCA platform can detect a high abundance of CTCs without EpCAM expression that was not able to be detected by CellSearch in SCLC^12^. Previous studies have reported similar findings, in which phenotypic variability of CTCs in patients with SCLC, with a specific subpopulation of CTCs being clinically relevant^17,27^. Furthermore, the single-cell prediction model supports the hypothesis of CTC phenotypic variability within SCLC. We hypothesized that the single-cell prediction model would use primarily CK expression as the main predictor input, but the Vim expression was also a top predictor supporting the importance of CTC heterogeneity in SCLC. As we have shown in SCLC^13^ and also from the other cancer types^28–30^, liquid biopsy approaches that are unbiased like the HDSCA platform will result in higher efficiency in isolating CTCs and detecting ultra-rare CTCs from SCLC patients.

Tumor plasticity enables a subset of cancer cells to transition between different cell states that accelerate tumor progression and metastasis^9,11,31^. The single-cell sequencing results confirm the existence of tumor cell plasticity by indicating that a phenotypically heterogeneous population of cells can be genomically stable. Cancer stem-like cells are a subset of cancer cells that have the ability to generate the intra-tumor heterogeneity of different cell phenotypes from differentiation^32,33^. EMT is one demonstration of tumor plasticity, with the intermediate states between the epithelial and mesenchymal phenotypes being associated with poor patient survival and chemotherapy resistance^8,32^. The CK CTCs and the CK|Vim CTCs harboring clonal alterations detected in this study potentially indicate the presence of EMT. Notably, other phenotypic rare cells were also identified with clonal alterations suggesting further dynamic cellular plasticity. Cellular plasticity is fundamental to SCLC tumorigenesis, thus requiring longitudinal prognostic tools to properly characterize the dynamic cell state. We have shown that a minimally invasive liquid biopsy which allows for repeated sampling can address the challenges associated with the detection of variable cell states with the evidence of clinical utility. Our results highlight the heterogeneity of circulating rare cells by the liquid biopsy approach with the identification of tumor cell plasticity. Further investigation is warranted to overcome the limitations of this study regarding the small sample size of enrolled patients and the number of characterized single cells.

In conclusion, in this study we establish the validity of circulating rare cell detection by stratifying SCLC patients and NDs with a high degree of accuracy using a classification model. Further, the data presented here provides evidence for cellular phenotypic plasticity, through the detection of heterogeneous circulating rare cells carrying the clonal tumor genotype with hallmarks of SCLC. Although matching tumor tissue was not available for direct genomic comparison, work by ourselves and others^22,23^ in other cancers have shown that clonal CTC populations closely reflect the genomics of clonal cells in the tumor. This provides new information for the potential stratification of treatments and the development of targeted therapeutics. This study demonstrates that liquid biopsy can provide a non-invasive route of tissue sampling with the opportunity for clinical monitoring and the development of better-stratified targeted therapies.

## Materials and methods

### Liquid biopsy samples

Patients with histological or cytological confirmation of chemotherapy-naive SCLC were recruited and consented at The Christie NHS Foundation Trust according to an ethically approved protocol (NHS Northwest 9 Research Ethical Committee). Informed written consent was obtained from all subjects. Experimental protocols were approved by the Institutional Review board of Clinical and Experimental Pharmacology Laboratory at the Paterson Institute for Cancer Research in Manchester. All methods were conducted in compliance with the guidelines of the International Conference on Harmonisation Good Clinical Practice (ICH Harmonised Tripartite Guideline E6: Note for Guidance on Good Clinical Practice (CPMP/ICH/135/95) Step 5), the Declaration of Helsinki, and in accordance with applicable regulations on the use of human tissue for research. Peripheral blood samples of up to 10 ml were collected at the entry to the study in blood collection tubes (Cell-free DNA, Streck, La Vista, NE, USA) and processed by the Convergent Science Institute in Cancer (CSI-Cancer) at the University of Southern California within 48 h as previously described^24^. In brief, after red blood cell lysis, nucleated cells were attached as a monolayer on custom-made glass slides (Marienfeld, Lauda, Germany) and cryopreserved until analysis. Peripheral blood samples from 10 NDs with no known pathology were collected from Scripps Research Institute and processed according to standard operating procedures.

### Immunofluorescence staining and imaging

Immunofluorescence staining was performed with the use of an IntelliPATH FLX™ autostainer (Biocare Medical LLC, Irvine, CA, USA) in batches of 50 slides with approximately 6 million nucleated cells as previously described^24,28^. Two assays were utilized in this study and are described below. Following immunofluorescence staining, slides were imaged using a custom-made fluorescent scanning microscope.

#### Landscape assay^28^

Two slides from each patient were stained with pan-cytokeratin (CK; clones: C-11, PCK-26, CY-90, KS-1A3, M20, A53-B/A2, C2562, Sigma, St. Louis, MO; CK19, clone: RCK108, GA61561-2, Dako, Carpinteria, CA; Alexa Fluor® 555 goat anti-mouse IgG1 secondary antibody, A21127, Invitrogen, Carlsbad, CA) to identify epithelial cells, a combination of CD31 (2.5 μg/ml; clone: WM59, MCA1738A647, BioRad, Hercules, CA) and CD45 (clone: F10-89-4, MCA87A647, AbD Serotec, Raleigh, NC) marking endothelial and immune cells, Vimentin (VIM; clone: D21H3, 9854BC, Cell Signaling, Danvers, MA) to identify mesenchymal cells, and DAPI (DAPI; D1306, ThermoFisher) marking the nucleus of the cell.

#### EpCAM-targeted assay

Two slides from patient UM-001 were stained with pan-cytokeratin (CK) and CD45 antibodies as described above with DAPI^24^ complemented with a monoclonal EpCAM antibody (1:250, 324202, Biolegend, San Diego, CA; Alexa Fluor® 488 goat anti-mouse IgG2b secondary antibody, 1:500, A21141, Invitrogen, Carlsbad, CA) at Epic Sciences. CTCs were identified with standard image analysis protocols^25–35^.

### Detection and classification of circulating rare cells

Detection of rare events from the imaging data set is conducted using an unsupervised clustering algorithm that clusters rare cells using extracted quantitative morphologic features, as previously reported^24,25^. The circulating rare cells detected were further classified into eight channel-based cell classifications defined by the fluorescence signal intensities and distribution of four different channel markers. For the Landscape assay, these groups include CK-positive only CTCs, CK|VIM-positive CTCs, CK|(CD45/CD31)-positive cells, CK|VIM|(CD45/CD31)-positive cells, DAPI-positive only cells, (CD45/CD31)-positive only cells, VIM-positive only cells, and VIM|(CD45/CD31)-positive cells. For the EpCAM-targeted assay, these groups include CK-positive only CTCs, CK|EpCAM-positive CTCs, CK|CD45-positive cells, EpCAM|CD45-positive cells, CK|EpCAM|CD45-positive cells, DAPI-positive only cells, CD45-positive only cells, and EpCAM-positive only cells. Rare cell channel-based classification was validated by a second trained analyst.

### Patient-level classification modeling

The morphologic features of the rare cells identified were used to map these events onto a pre-constructed t-SNE of previously identified rare cells from various cancer types and NDs. Based on the nearest representative cell within the multi-dimensional t-SNE space, the cells were then assigned a cell identifier. Using the morphological hierarchy of the representative cells, the identified rare events for the SCLC patients and NDs can be clustered into similar groups. Using a top-down approach and starting with two clusters, the counts of cells per mL of each group are calculated for both SCLC patients and NDs. These counts are used as input data for a random forest classification model of 1000 decision trees to predict whether the distribution depicts SCLC patients or NDs. This process is repeated with 3 clusters up until a predefined stopping criteria (e.g., 100 clusters). The optimal number of cell clusters was determined by the minimum out of bag (OOB) error rate of the random forest model. OOB error rate is calculated during the training process as the random forest will randomly hold out a small subset of input data to be used as testing data on each of the decision trees that are constructed. This method of measuring model performance was chosen due to the small number of SCLC patients and NDs available. Using the feature importance of the optimal random forest model, the cell clusters were ordered to identify those that contribute the most to correctly stratifying the classes. Next, starting with the top two most important clusters, random forest models were incrementally recreated to determine the best model with the lowest error rate as a means of pruning the final input dataset (i.e., feature reduction). To more easily visualize the cells within each cluster, we organize the events by their morphological hierarchy and display the events in a grid-like fashion.

### Whole genome single cell copy number alteration

Rare cell relocation, re-imaging, isolation, next-generation sequencing (NGS), and CNA analysis were conducted as previously reported^29,36–38^. In brief, cells of interest were relocated using registered coordinates and imaged with a 40× objective. Subsequently, individual cells were extracted from slides using a robotic micromanipulator system followed by single-cell whole genome amplification (WGA; Sigma-Aldrich; Cat# WGA4). Libraries were constructed using the DNA Ultra Library Prep Kit (New England Biolabs; Cat# E7370) and sequenced using Illumina NextSeq 500 at USC Genomics Core or at Fulgent Genomics (Temple City, CA). The copy number profile of each individual cell was reconstructed from the frequency of unique reads mapped to the human genome (hg19). Only cells with total reads above 50,000 per cell, a total alignment rate above 50%, minimal noise, an in-house quality score greater than or equal to 2.5, and had reads across the whole genome (no apoptosis-induced alterations) were included in the analysis.

### Single-cell alteration classification model

To investigate the relationship between phenotype and genotype in SCLC, multiple predictive models (e.g. random forest, naïve Bayes, and support vector machine) were implemented. The morphometric features from the HDSCA image data of the Landscape assay-stained cells were the input parameters. A binary “Clonally Altered” vs. “Not Clonally Altered” designation for each individual cell genomic profile was the target output. A trained genomic analyst provided guidance on input selection for training according to the genomic instability, eliminating genomic profiles exhibiting technical artifacts, and clonality as determined by the presence of more than two cells having at least three alterations in concordance from the same sample. Feature selection was conducted to prevent overfitting of the data and issues with multicollinearity, as well as to further optimize the model. Features with a correlation above 0.9 were grouped together and the feature with the highest variance was selected to represent the subset, resulting in a final set of 56 features.

### Statistical analysis

Statistical analyses and visualization were performed with R (version 4.1.2). Statistical significance was determined at a *p-value* ≤ 0.05. Mann–Whitney U test was conducted to observe the statistical differences between SCLC patients and NDs. Prediction accuracy was measured by the AUC score.

## Supplementary Information

Supplementary Information 1.

Supplementary Table S1.

## Data Availability

All data discussed in this manuscript are included in the main manuscript text or supplementary materials. The imaging data are available through the BloodPAC Data Commons, Accession ID “BPDC000130” (https:/data.bloodpac.org/discovery/BPDC000130/).

## References

[1] Siegel RL, Miller KD, Fuchs HE, Jemal A (2022). Cancer statistics, 2022. CA Cancer J. Clin..

[2] Rudin CM, Brambilla E, Faivre-Finn C, Sage J (2021). Small-cell lung cancer. Nat. Rev. Dis. Primer.

[3] Foy V, Fernandez-Gutierrez F, Faivre-Finn C, Dive C, Blackhall F (2017). The clinical utility of circulating tumour cells in patients with small cell lung cancer. Transl. Lung Cancer Res..

[4] Taniguchi H, Sen T, Rudin CM (2020). Targeted therapies and biomarkers in small cell lung cancer. Front. Oncol..

[5] Mathieu L (2021). FDA approval summary: Atezolizumab and durvalumab in combination with platinum-based chemotherapy in extensive stage small cell lung cancer. Oncologist.

[6] Sabari JK, Lok BH, Laird JH, Poirier JT, Rudin CM (2017). Unravelling the biology of SCLC: Implications for therapy. Nat. Rev. Clin. Oncol..

[7] Krohn A, Ahrens T, Yalcin A, Plö-Nes T, Wehrle J (2014). Tumor cell heterogeneity in small cell lung cancer (SCLC): Phenotypical and functional differences associated with epithelial-mesenchymal transition (EMT) and DNA methylation changes. PLoS ONE.

[8] Bakir B, Chiarella AM, Pitarresi JR, Rustgi AK (2020). EMT, MET, plasticity, and tumor metastasis. Trends Cell Biol..

[9] Meacham CE, Morrison SJ (2013). Tumour heterogeneity and cancer cell plasticity. Nature.

[10] Ruan J (2021). Tumor cell plasticity and intrinsic immunogenicity: Implications for immunotherapy resistance in small-cell lung cancer. Thorac. Cancer.

[11] da Silva-Diz V, Lorenzo-Sanz L, Bernat-Peguera A, Lopez-Cerda M, Muñoz P (2018). Cancer cell plasticity: Impact on tumor progression and therapy response. Semin. Cancer Biol..

[12] Williamson SC (2016). Vasculogenic mimicry in small cell lung cancer. Nat. Commun..

[13] Basumallik, N. & Agarwal, M. Small cell lung cancer. In *StatPearls* (StatPearls Publishing, 2022). 29494065

[14] Pizzutilo EG (2021). Liquid biopsy for small cell lung cancer either de novo or transformed: Systematic review of different applications and meta-analysis. Cancers.

[15] Revelo AE (2019). Liquid biopsy for lung cancers: An update on recent developments. Ann. Transl. Med..

[16] Rolfo C, Russo A (2020). Liquid biopsy for early stage lung cancer moves ever closer. Nat. Rev. Clin. Oncol..

[17] De Luca A, Gallo M, Esposito C, Morabito A, Normanno N (2021). Promising role of circulating tumor cells in the management of SCLC. Cancers.

[18] Setayesh SM, Hart O, Naghdloo A (2022). Multianalyte liquid biopsy to aid the diagnostic workup of breast cancer. NPJ Breast Cancer.

[19] George J (2015). Comprehensive genomic profiles of small cell lung cancer. Nature.

[20] Hu J (2019). Comprehensive genomic profiling of small cell lung cancer in Chinese patients and the implications for therapeutic potential. Cancer Med..

[21] Sozzi G (1996). The FHIT gene at 3p142 is abnormal in lung cancer. Cell.

[22] Welter L (2020). Treatment response and tumor evolution: Lessons from an extended series of multianalyte liquid biopsies in a metastatic breast cancer patient. Cold Spring Harbor Mol. Case Stud..

[23] Riebensahm C, Joosse SA, Mohme M (2019). Clonality of circulating tumor cells in breast cancer brain metastasis patients. Breast Cancer Res.

[24] Marrinucci D (2012). Fluid biopsy in patients with metastatic prostate, pancreatic and breast cancers. Phys. Biol..

[25] Scher HI (2016). Association of AR-V7 on circulating tumor cells as a treatment-specific biomarker with outcomes and survival in castration-resistant prostate cancer. JAMA Oncol..

[26] Hodgkinson CL (2014). Tumorigenicity and genetic profiling of circulating tumor cells in small-cell lung cancer. Nat. Med..

[27] Messaritakis I (2017). Phenotypic characterization of circulating tumor cells in the peripheral blood of patients with small cell lung cancer. PLoS ONE.

[28] Chai S (2021). Platelet-coated CTC as a predictive biomarker of mCRPC platelet-coated circulating tumor cells are a predictive biomarker in patients with metastatic castrate resistant prostate cancer. Mol. Cancer.

[29] Shishido SN (2022). Characterization of cellular and acellular analytes from pre-cystectomy liquid biopsies in patients newly diagnosed with primary bladder cancer. Cancers.

[30] Kolenčík D (2020). Liquid biopsy in colorectal carcinoma: Clinical applications and challenges. Cancers.

[31] Shen S, Clairambault J (2020). Cell plasticity in cancer cell populations. F1000Research.

[32] Wahl GM, Spike BT (2017). Cell state plasticity, stem cells, EMT, and the generation of intra-tumoral heterogeneity. NPJ Breast Cancer.

[33] Thankamony AP, Saxena K, Murali R, Jolly MK, Nair R (2020). Cancer stem cell plasticity—a deadly deal. Front. Mol. Biosci..

[34] Scher HI (2018). Assessment of the validity of nuclear-localized androgen receptor splice variant 7 in circulating tumor cells as a predictive biomarker for castration-resistant prostate cancer. JAMA Oncol..

[35] Armstrong AJ (2019). Prospective multicenter validation of androgen receptor splice variant 7 and hormone therapy resistance in high-risk castration-resistant prostate cancer: The PROPHECY study. J. Clin. Oncol..

[36] Dago AE (2014). Rapid phenotypic and genomic change in response to therapeutic pressure in prostate cancer inferred by high content analysis of single circulating tumor cells. PLoS ONE.

[37] Ruiz C (2015). Limited genomic heterogeneity of circulating melanoma cells in advanced stage patients. Phys. Biol..

[38] Malihi PD (2020). Single-cell circulating tumor cell analysis reveals genomic instability as a distinctive feature of aggressive prostate cancer. Clin. Cancer Res..

