# Supplementary material for: Plasticity of circulating tumor cells in small cell lung cancer
Source: Sci Rep. 2023 Jul 21;13:11775. doi: 10.1038/s41598-023-38881-5 (PMC10362013; doi:10.1038/s41598-023-38881-5)

**Supplementary Information**


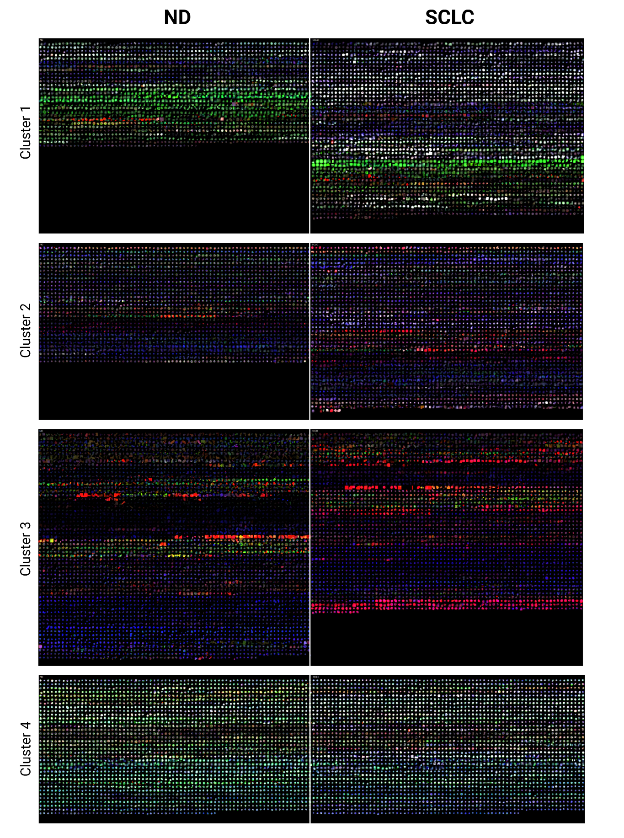


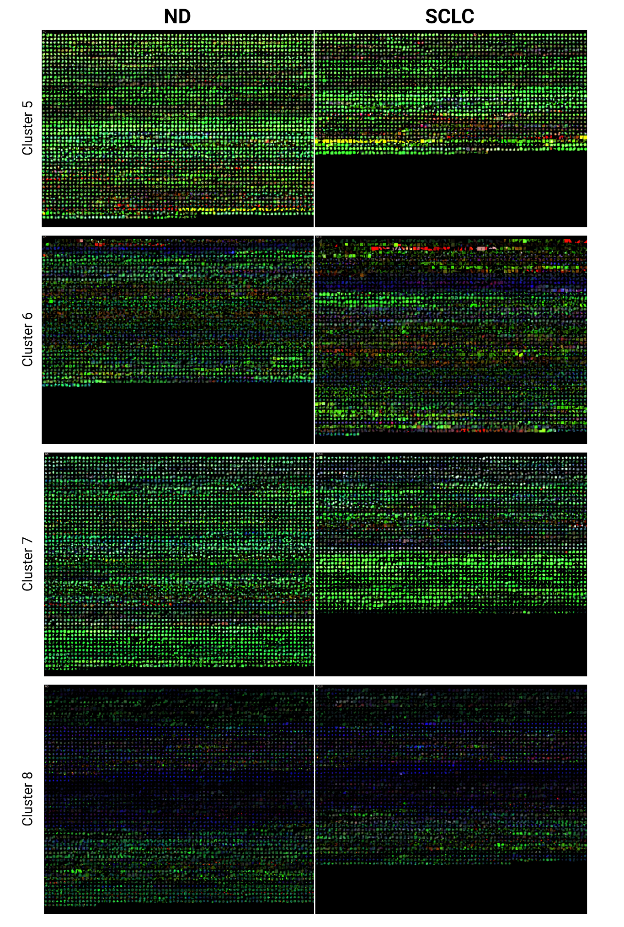

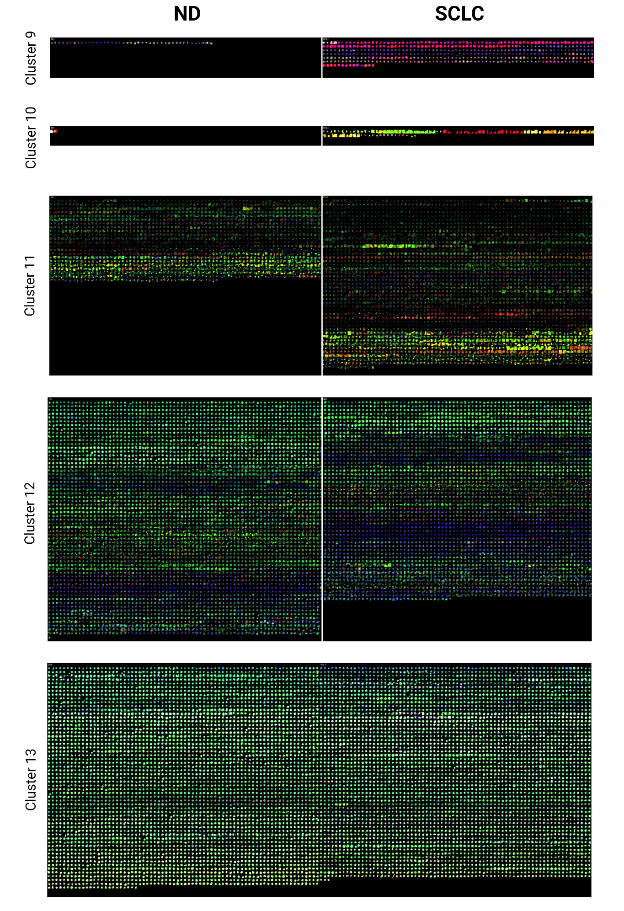

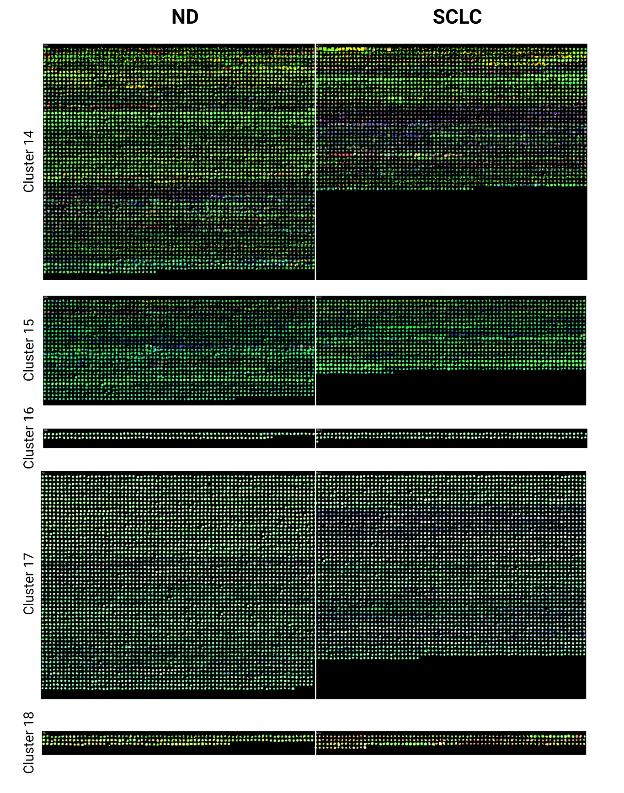

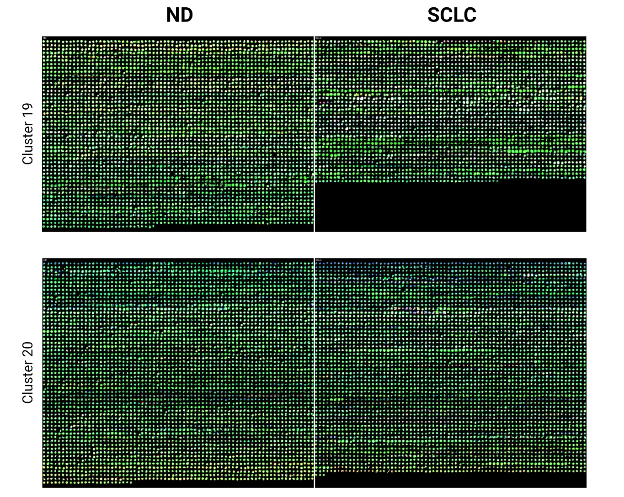


**Figure S1: Representative cell images of the top important clusters**. The cells of SCLC patients are shown on the right side and the cells of NDs are shown on the left side for each cluster.


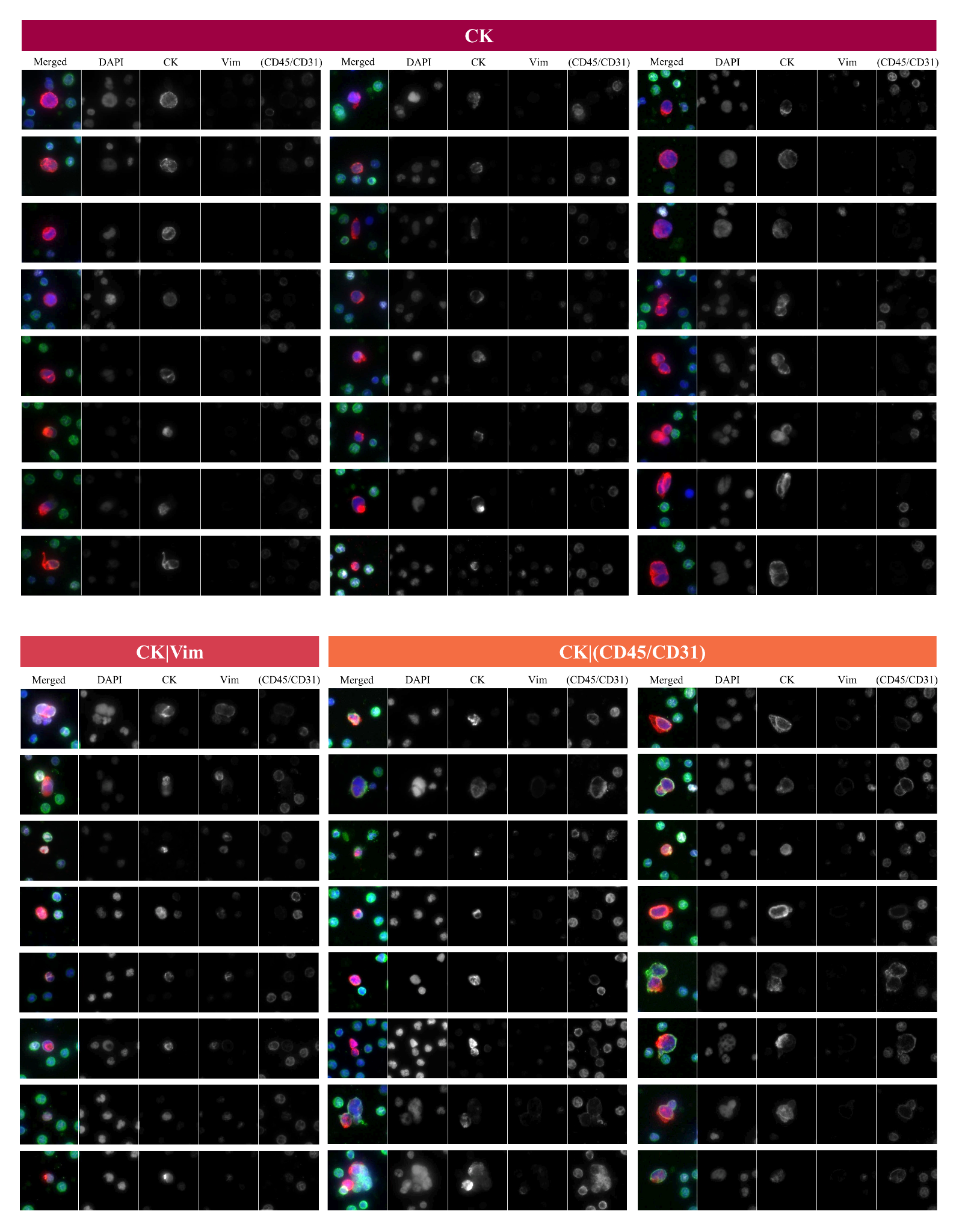


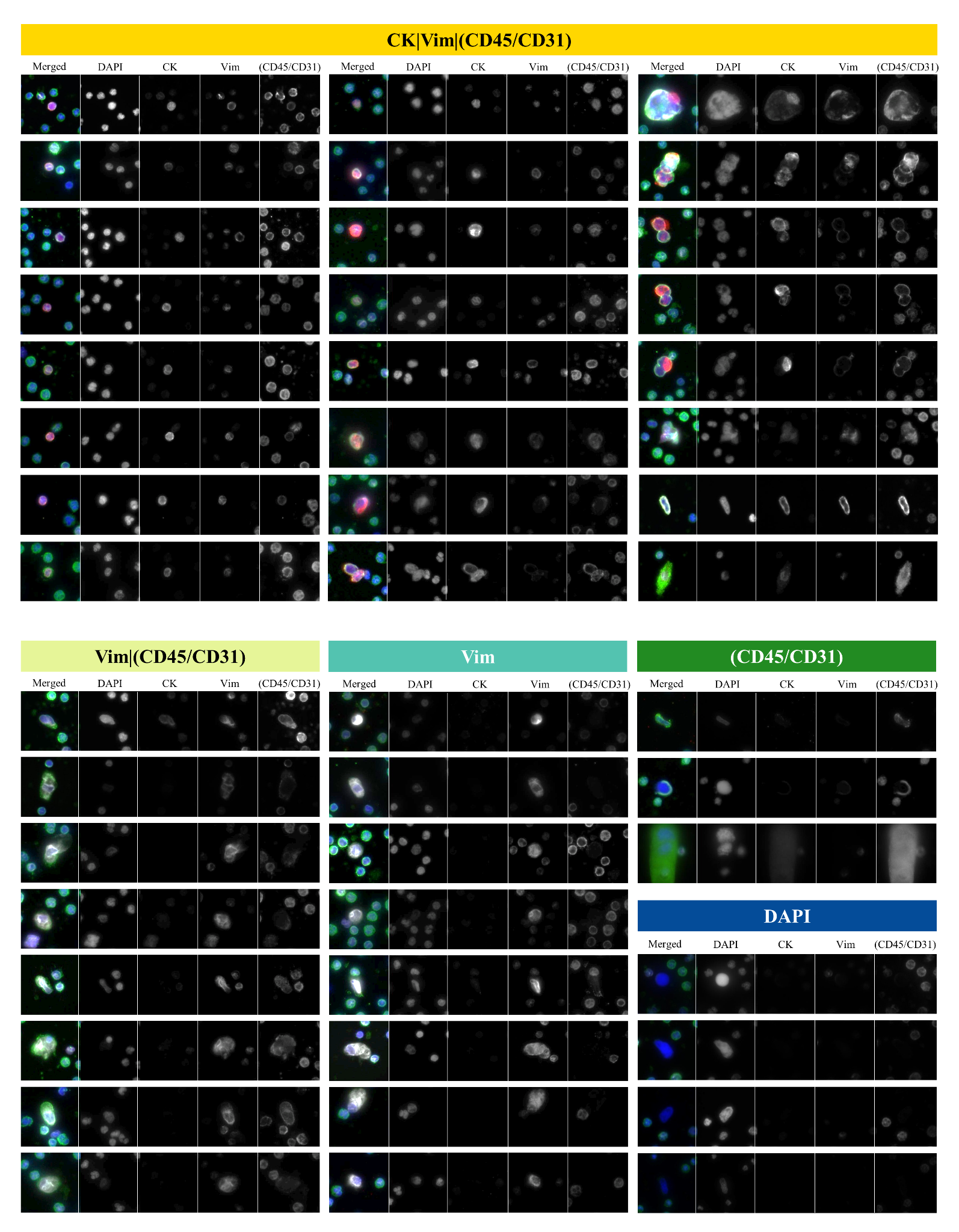


**Figure S2.** **Circulating rare cells identified in the peripheral blood from SCLC patient samples using HDSCA3.0 with the Landscape assay.** **A.** Representative cell images of each cell group. Each row shows a composite image plus each of the four channels separately; DAPI in blue, CK in red, Vim in white, (CD45/CD31) in green


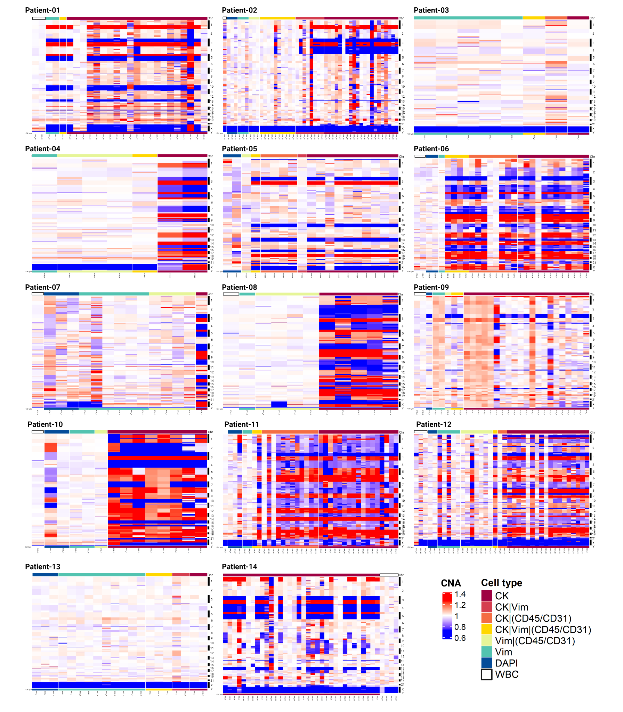


**Figure S3: Single cell CNA profiling of rare cells detected in peripheral blood samples from 14 SCLC patients.** CNA heatmap of all sequenced cells from 14 SCLC patients. The rare cell type of each cell is annotated with color labels at the bottom of each heatmap. The color labels for each rare cell type are described at the bottom right. CNA gains are shown in red, neutrals in white and losses in blue.

**Table S1: The number of circulating rare cells identified in the peripheral blood from each ND sample.**

**
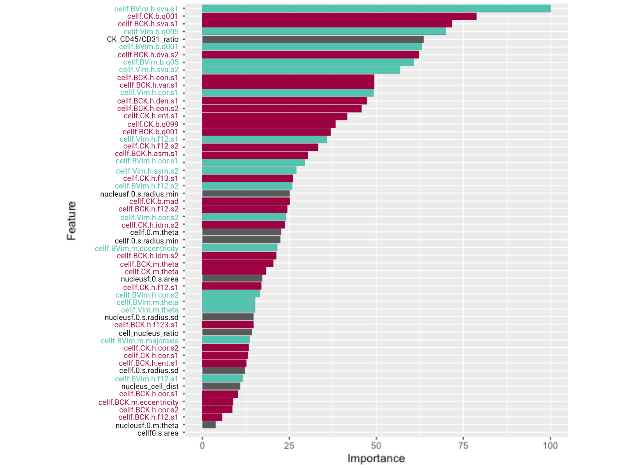
**

**Figure S4: Feature importance of the features for the random forest model.**


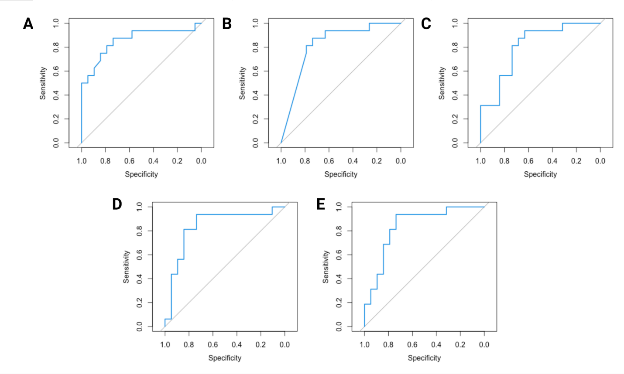


**Figure S5: Receiver operator characteristic curve of each model with all features. A.** Random Forest **B.** Naïve Bayes **C.** Support vector machine linear **D.** Support vector machine radial **E.** Support vector machine poly.


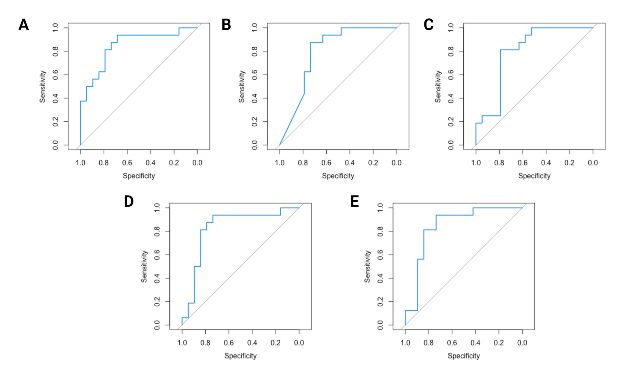


**Figure S6: Receiver operator characteristic curve of each model with selected features. A.** Random Forest **B.** Naïve Bayes **C.** Support vector machine linear **D.** Support vector machine radial **E.** Support vector machine poly.

**Table S2: Performance of all models.** All models are performed with and without feature selection.


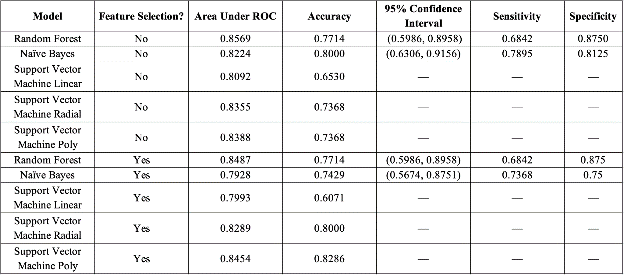

Supplement: Supplementary file 1 — Supplementary Information 1. [file 41598_2023_38881_MOESM1_ESM.docx]
